# A Novel Systemic Inflammation Prognostic Score to Stratify Survival in Elderly Patients With Cancer

**DOI:** 10.3389/fnut.2022.893753

**Published:** 2022-07-05

**Authors:** Guo-Tian Ruan, Hai-Lun Xie, Li Deng, Yi-Zhong Ge, Qi Zhang, Zi-Wen Wang, Xi Zhang, He-Yang Zhang, Meng Tang, Meng-Meng Song, Xiao-Wei Zhang, Ming Yang, Lei Pan, Kun-Hua Wang, Ming-Hua Cong, Yi-Zhen Gong, Meng-Yan Wang, Han-Ping Shi

**Affiliations:** ^1^Department of Gastrointestinal Surgery, Beijing Shijitan Hospital, Capital Medical University, Beijing, China; ^2^Department of Clinical Nutrition, Beijing Shijitan Hospital, Capital Medical University, Beijing, China; ^3^Key Laboratory of Cancer FSMP for State Market Regulation, Beijing, China; ^4^Department of Respiratory and Critical Care Medicine, Beijing Shijitan Hospital, Capital Medical University, Beijing, China; ^5^Yunnan University, Kunming, China; ^6^General Surgery Clinical Medical Center of Yunnan Province, Kunming, China; ^7^Comprehensive Oncology Department, National Cancer Center/Cancer Hospital, Chinese Academy of Medical Sciences and Peking Union Medical College, Beijing, China; ^8^Department of Gastrointestinal Surgery, Guangxi Medical University Cancer Hospital, Nanning, China; ^9^Guangxi Clinical Research Center for Colorectal Cancer, Nanning, China; ^10^Department of General Surgery, The 906th Hospital of the Joint Logistics Support Force of the Chinese People’s Liberation Army, Ningbo, China

**Keywords:** SIPS, systemic inflammation, malnutrition, elderly patients with cancer, overall survival

## Abstract

**Background:**

Elderly patients with cancer face the challenge of systemic inflammation, which can lead to a poor prognosis. Existing inflammatory indices cannot fully reflect the immune-inflammatory status of patients. This study aimed to develop a new scoring system to predict the survival of elderly patients with cancer using inflammatory indices, namely, the systemic inflammation prognostic score (SIPS).

**Materials and Methods:**

This prospective multicenter study included a total of 1,767 patients with cancer, with a mean age of 70.97 ± 5.49 years, of whom 1,170 (66.2%) were men. We performed the least absolute shrinkage and selection operator (LASSO) regression to screen inflammatory indicators to include in constructing SIPS. Prognostic analysis of SIPS was performed using univariate and multivariate survival analyzes. The prognostic value of SIPS and its components were compared using the prognostic receiver operating characteristic curve and concordance index. The population was divided into the training cohort and the validation cohort in a 7:3 ratio and a SIPS prognostic analysis was performed.

**Results:**

The LASSO regression selected C-reactive protein (CRP) (≤ 9.81, “0”; > 9.81, “1”), geriatric nutritional risk index (GNRI) (≤ 93.85, “1”; 93.85, “0”), advanced lung cancer inflammation index (ALI) (≤ 23.49, “1”; > 23.49, “0”), and lymphocyte to C-reactive protein ratio (LCR) (≤ 2523.81, “1”; > 2523.81, “0”) to develop SIPS. Patients were divided into the three groups based on the total SIPS: low-risk (0), moderate-risk (1–2), and high-risk (3–4). On the multivariate survival analysis, patients in the moderate-risk [*P* < 0.001, hazard ratio (HR) = 1.79, 95% CI: 1.47–2.17] and high-risk groups (*P* < 0.001, HR = 2.40, 95% CI: 1.98–2.92) showed a worse prognosis than those in the low-risk group. The total cohort, training cohort, and validation cohort all showed that SIPS had better survival prediction than CRP, GNRI, ALI, and LCR. The HRs were 2.81 times higher in patients in the high-risk group with malnutrition than in patients in the low-risk group without malnutrition.

**Conclusion:**

SIPS was an independent prognostic indicator in elderly patients with cancer. Malnutrition in the high-risk group increased the mortality risk.

## Introduction

The International Agency for Research on Cancer has updated its 2020 global cancer incidence and mortality estimates, showing an estimated 19.3 million new cancers and 10 million cancer-related deaths in 2020 (1). Notably, approximately half of the cases and 58.3% of cancer deaths occurred in Asia (1). The aging population has led to an increase in new cancer cases worldwide. In 2018, there were more than 9 million cancer cases in adults aged 65 years and older worldwide and this is expected to increase to nearly 14 million cases by 2040 (2). Functional alterations, age, and polydrug comorbidities make treatment-related symptoms challenging in elderly patients with cancer (3). Cancer cachexia is very common in the elderly oncological population and is associated with functional impairment (4).

A hallmark of aging is the presence of chronic low-grade inflammation characterized by elevated levels of interleukin-6 (IL-6), tumor necrosis factor-α (TNF-α), and C-reactive protein (CRP) (5). Cancer-related inflammation is the seventh hallmark of cancer (6). In recent years, there has been increasing evidence that aging plays an important role in the triangular relationship between nutrition, inflammation, immunity, and cancer (7). Inflammation promotes tumor initiation, staging, and progression (8). In the tumor microenvironment, inflammation contributes to the proliferation and survival of malignant cells, angiogenesis, metastasis, disruption of adaptive immunity, reduced response to hormones, and chemotherapeutic drugs (6). In patients diagnosed with cancer, both increased local immune cell infiltration in tumors and elevated systemic inflammatory responses may be important indicators of cancer progression and prognosis (8). Biomarkers in preoperative or pretreatment peripheral blood reflect the patient’s baseline inflammatory and immune status to a certain extent and are considered potential markers for predicting prognosis due to their high accessibility in clinical practice (9). Systemic inflammation can be assessed by various biochemical or blood markers routinely measured in routine blood tests or by ratios derived from these markers (10), such as CRP (11), neutrophil-to-lymphocyte ratio (NLR) (9), geriatric nutritional risk index (GNRI) (12), and advanced lung cancer inflammation index (ALI) (13). Some studies have combined known prognostic factors to establish new scoring systems to predict prognosis and guide clinical practice, such as the lymphocyte CRP score (14) and the Controlling Nutritional Status score (15). However, markers commonly used in clinical practice are not comprehensively utilized in elderly patients with cancer, and we believe that combining these markers can predict clinical survival more accurately than using a single marker.

This study aimed to develop a novel prognostic scoring system, named the systemic inflammation prognostic score (SIPS), based on inflammation-related clinical parameters recorded in our prospective multicenter cohort, to improve survival prediction in elderly patients with cancer, and to investigate inflammation and nutritional effects on poor prognosis in these patients.

## Materials and Methods

### Study Subjects

This prospective multicenter cohort study included patients with cancer aged ≥ 18 years in multiple Chinese medical institutions between July 2013 and June 2021. The inclusion criteria for this study were as follows: (1) age at least 18 years old; (2) pathologically diagnosed with cancer; and (3) conscious and able to answer questions independently. There are no strict exclusion criteria. A total of 5,221 patients with cancer with complete information were reviewed, of whom 1,767 elderly patients with cancer (age ≥ 65 years) were included in the final study (Supplementary Figure 1). This study protocol was conducted by the Declaration of Helsinki and this study was approved by the ethical review committee of the participating institutions (registration number: ChiCTR1800020329). Written informed consent was obtained from all the patients.

### Data Collection and Definition of Variables

General clinical data were collected from patients’ electronic hospital records and questionnaires of interviews performed by experienced medical personnel. These included age, sex, body mass index (BMI), lifestyle (smoking, yes vs. no; alcohol consumption, yes vs. no), cancer-related data (tumor stage; surgical treatment, yes vs. no; chemotherapy, yes vs. no; radiotherapy, yes vs. no; and immunotherapy, yes vs. no), nutrition-related indicators [Scored Patient-Generated Subjective Global Assessment (PG-SGA) tool; nutritional intervention, yes vs. no;], quality of life and performance status assessment [European Organization for Research and Treatment of Cancer Quality of Life Questionnaire-Core 30 (EORTC QLQ-C30) and the Karnofsky Performance Status (KPS)], and laboratory blood test indicators. BMI (kg/m^2^) was calculated as the ratio of weight to height squared and was divided into four categories according to Chinese population classification standards: underweight (< 18.5), normal weight (18.5–23.9), overweight (24–27.9), and obese (>28). The tumor stage was assessed according to the tumor, necrosis, and metastasis (TNM) Classification of Malignant Tumors, 8th edition. Malnutrition diagnosed using the PG-SGA was classified into three nutritional states: severe malnutrition, moderate malnutrition, and good nutrition (16).

### Assessment of Inflammatory Markers

Blood samples were collected to measure serum markers, namely, CRP, neutrophils (N), lymphocytes (L), platelets (P), glucose (Glu), albumin (ALB), and globulin (GLB). These baseline inflammatory markers constituted the following ratios and indices: NLR (N/L ratio) (13), PLR (P/L ratio) (13), prognostic nutrition index (PNI), 10 × ALB + 0.005 × L) (13), systemic immune-inflammation index (SII), P × NLR) (13), ALI (BMI × ALB/NLR) (13), CAR (CRP/ALB ratio) (17), AGR (ALB/GLB ratio) (18), LCR (L/CRP ratio) (14), GNRI (1.489 × ALB + [Present body weight (PBW)/Ideal body weight (IBW)]) (12), mGNRI (modified GNRI, 1.489 × CRP + 41.7 × PBW/IBW) (12), and nutritional risk index (NRI), 1.519 × ALB + 41.7 × PBW/IBW) (19). IBW was evaluated using the Lorentz equation: men = height (cm) − 100 − [(height − 150)/4]; women = height (cm) − 100 − [(height − 150)/2.5] (20).

### Outcomes

The primary endpoint observed in this study was overall survival (OS). OS was defined as the time from diagnosis of cancer to the time of death, loss of follow-up, or review of the last follow-up date.

### Statistical Analysis

For categorical variables, data are expressed as absolute frequencies and percentages and for continuous variables, data are expressed as means and SDs or medians and interquartile ranges. Categorical variables were analyzed using the chi-square test or Fisher’s exact test where appropriate and continuous variables were analyzed by using the Student’s *t*-test. Due to multicollinearity, we used the least absolute shrinkage and selection operator (LASSO) Cox regression model for dimensionality reduction, selecting the most optimal prognostic features from all the available inflammatory and relevant biomarkers (CRP, N, L, P, Glu, ALB, GLB, NLR, PLR, PNI, SII, ALI, CAR, AGR, LCR, GNRI, mGNRI, and NRI). The Pearson correlation analysis was used to estimate the correlation coefficients among inflammatory prognostic factors, when coefficients |*R*| > 0.4 and *P* < 0.05 were significantly correlated. We constructed SIPS from unrelated factors. The optimal truncation values of selected inflammatory parameters in elderly patients with cancer in this study were obtained based on the “survminer” package of the R platform. The cutoff values of ALI, GNRI, LCR, and CRP were 23.49, 93.85, 2523.81, and 9.81, respectively (Supplementary Figure 2 and Supplementary Table 1). We defined the following: CRP ≤ 9.81 was scored as “0,” CRP > 9.81 was scored as “1”; ALI ≤ 23.49 was scored as “1,” ALI > 23.49 was scored as “0”; GNRI ≤ 93.85 was scored as “1,” GNRI > 93.85 was scored as “0”; and LCR ≤ 2523.81 was scored as “1,” LCR > 2523.81 was scored as “0.” Patients were divided into the three groups based on the total SIPS, using a 4-point system, as follows: “0,” low-risk group; “1” or “2,” moderate-risk group; and “3” or “4,” high-risk group. The Kaplan–Meier method and log-rank test were used to produce the survival curves. The univariate and multivariate analyzes were performed using the Cox proportional risk model to determine the independent prognostic factors. Hazard ratios (HRs) and 95% CIs were used to assess the risk of death in patients. In the multivariable adjustment model, we constructed the following adjustment models: model 0, non-adjustment model; model 1, adjusted for age, sex, tumor stage, and BMI; and model 2, adjusted for age, sex, tumor stage, BMI, tumor type, smoking, drinking, KPS, surgery, radiotherapy, chemotherapy, immunotherapy, nutritional intervention, and EORTC QLQ-C30. We compared the prognostic value and discriminative ability of the SIPS inflammatory prognostic model with ALI, GNRI, LCR, and CRP using the prognostic receiver operating characteristic (ROC) curve and Harrell’s concordance index (C-index). Additionally, patients were randomly assigned to two independent cohorts (the training and validation cohorts) at a ratio of 7:3, and the prognostic value of SIPS was analyzed. All the statistical tests were two-tailed and statistical significance was inferred when the *P* value was < 0.05. Statistical analysis was performed using R Language 4.0.3^1^. The R packages included: “glmnet,” “foreign,” “survival,” “survminer,” “ggplot2,” “GGally,” and “timeROC.”

## Results

### Baseline Characteristics

The baseline characteristics of the patients are shown in Table 1. The median follow-up time was 32.3 (95% CI: 29.9–35.1) months. The mean age of patients was 70.97 ± 5.49 years, 1,170 (66.2%) were men, and 761 patients (43.1%) were ≥ 70 years old.

**TABLE 1:** Baseline characteristics of the study population.

Characteristics	Overall patients (n = 1767)
**Sex (%)**	
Male	1170 (66.2)
Female	597 (33.8)
Age [mean (SD)]	70.97 (5.49)
Age, > 70 years (%)	761 (43.1)
BMI [mean (SD)]	22.26 (3.52)
**BMI, kg/m^2^ (%)**	
<18.5	260 (14.7)
18.5**–**23.9	963 (54.5)
24**–**27.9	447 (25.3)
≥28	97 (5.5)
Smoking, yes (%)	917 (51.9)
Alcohol, yes (%)	427 (24.2)
**Tumor types (%)**	
Lung cancer	650 (36.8)
Gastric cancer	259 (14.7)
Esophageal cancer	139 (7.9)
Colorectal cancer	310 (17.5)
Other digestive cancers	138 (7.8)
Breast cancer	71 (4.0)
Female reproductive cancer	30 (1.7)
Urological cancer	87 (4.9)
Nasopharyngeal cancer	24 (1.4)
Other cancer	59 (3.3)
**Tumor stage (%)**	
I	129 (7.3)
II	290 (16.4)
III	479 (27.1)
IV	869 (49.2)
Surgery, yes (%)	762 (43.1)
Radiotherapy, yes (%)	154 (8.7)
Chemotherapy, yes (%)	950 (53.8)
Immunotherapy, yes, (%)	74 (4.2)
PGSGA [mean (SD)]	7.33 (5.02)
**PGSGA (%)**	
Well nourished	536 (30.3)
Malnourished	1231 (69.7)
Nutritional intervention, yes (%)	400 (22.6)
EORTCQLQ-C30 [mean (SD)]	48.07 (11.11)
KPS [mean (SD)]	82.37 (14.22)
KPS, < 60 (%)	169 (9.6)
Albumin, g/dl [mean (SD)]	38.11 (4.98)
Globulin, g/dl [mean (SD)]	30.08 (5.63)
Cholesterol, mmol/L [mean (SD)]	4.55 (1.11)
CRP, mg/L [median (IQR)]	19.14 (32.71)
Blood glucose, mmol/L [mean (SD)]	5.91 (1.87)
Neutrophil, *10^9^/L [mean (SD)]	4.49 (2.80)
Lymphocyte, *10^9^/L [mean (SD)]	1.53 (0.82)
Platelet [mean (SD)]	225.52 (90.64)
NLR [median (IQR)]	2.67 (2.39)
PLR (mean (SD)]	177.93 (132.23)
GLR [mean (SD)]	4.93 (3.85)
**PGSGA (%)**	
ALI [mean (SD)]	39.09 (34.33)
SII [median (IQR)]	577.93 (660.41)
CAR (median (IQR))	0.14 (0.50)
GNRI [mean (SD)]	95.77 (8.91)
mGNRI [mean (SD)]	44.96 (11.91)
NRI [mean (SD)]	96.91 (9.04)
AGR [mean (SD)]	1.31 (0.31)
PNI [mean (SD)]	45.74 (6.91)
LCR [median (IQR)]	2586.02 (5695.02)

SD: standard deviation; IQR: interquartile range; BMI: body mass index; CRP: C-reactive protein; NLR: neutrophil to lymphocyte ratio; PLR: platelet to lymphocyte ratio; GLR, glucose to lymphocyte ratio; ALI: advanced lung cancer inflammation index; SII: systemic immune inflammation index; CAR: C-reactive protein to albumin ratio; GNRI: geriatric nutrition risk index; mGNRI: modified geriatric nutrition risk index; AGR: albumin to globulin ratio; PNI: Prognostic Nutritional Index; NRI: nutrition risk index; LCR: lymphocyte to C-reactive protein ratio; ECOG PS: eastern cooperative oncology group performance status; KPS: karnofsky performance status; PGSGA: patient-generated subjective global assessment; EORTC QLQ-C30: The European Organization for Research and Treatment of Cancer (EORTC), Quality of Life Questionnaire-Core 30 (QLQ-C30). “*” means “multiply.”

### Feature Selection and Analysis and Construction of Inflammatory Index Models

**FIGURE 1:**
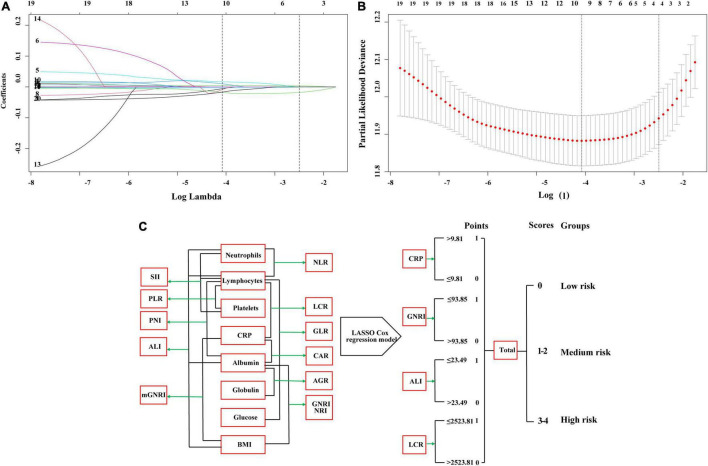
The least absolute shrinkage and selection operator (LASSO) Cox regression model screening parameters, SIPS construction process, and risk stratification. **(A,B)** The LASSO Cox regression model screening parameters; **(C)** SIPS construction process and risk stratification. SIPS: systemic inflammation prognostic score; BMI: body mass index; CRP: C-reactive protein; NLR: neutrophil to lymphocyte ratio; PLR: platelet to lymphocyte ratio; GLR, glucose to lymphocyte ratio; ALI: advanced lung cancer inflammation index; SII: systemic immune inflammation index; CAR: C-reactive protein to albumin ratio; GNRI: geriatric nutrition risk index; mGNRI: modified geriatric nutrition risk index; AGR: albumin to globulin ratio; PNI: Prognostic Nutritional Index; NRI: nutrition risk index; LCR: lymphocyte to C-reactive protein ratio.

**FIGURE 2:**
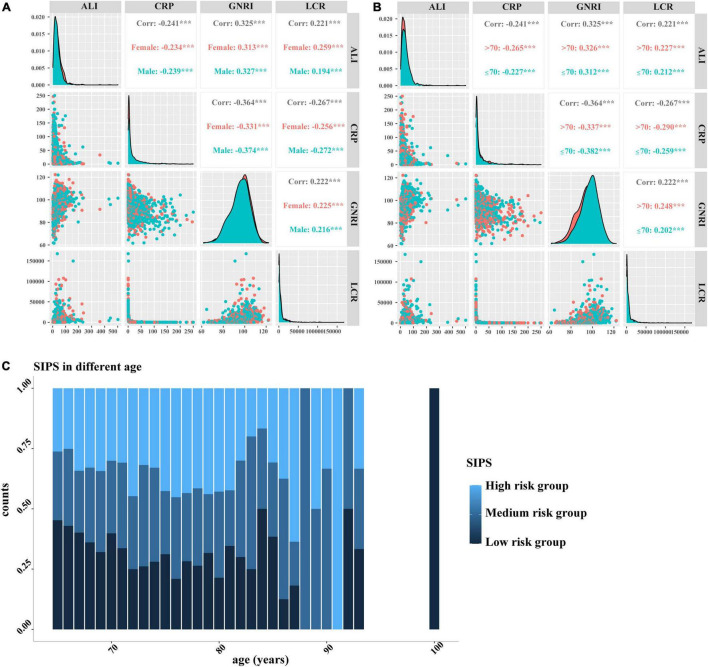
The Pearson correlation analysis and distribution of SIPS by age. **(A–B)** The Pearson correlation analysis among CRP, GNRI, ALI, and LCR. **(A)** Stratified by sex; **(B)** Stratified by age; and **(C)** distribution of SIPS by age. SIPS: systemic inflammation prognostic score; CRP: C-reactive protein; GNRI: geriatric nutrition risk index; ALI: advanced lung cancer inflammation index; LCR: lymphocyte to C-reactive protein ratio.

We performed the LASSO regression to select prognostic indices corresponding to the optimal value of λ0.1se = 0.082. Four variables with non-zero coefficients were retained in the LASSO analysis: CRP, GNRI, ALI, and LCR (Figure 1). The Pearson coefficient analysis showed that CRP, GNRI, ALI, and LCR were not significantly correlated with each other. Similarly, the results of the correlation analyzes were consistent in both the sex and age subgroups and no significant association was observed (Figures 2A, B).

We analyzed the prognostic value of the individual biomarkers in elderly patients with cancer and survival curve results showed that patients with high CRP, low GNRI, high ALI, and low LCR had worse OS than those with low CRP, high GNRI, high ALI, and high LCR (all *P* < 0.001) (Supplementary Figure 3). The multivariate survival analysis showed that high CRP (model 2: *P* < 0.001, HR = 1.67, 95% CI: 1.44–1.92), low GNRI (model 2: *P* < 0.001, HR = 1.67, 95% CI: 1.44–1.94), low ALI (model 2: *P* < 0.001, HR = 1.57, 95% CI: 1.35–1.82), and low LCR (model 2: *P* < 0.001, HR = 1.69, 95% CI: 1.46–1.96) were significantly associated with shorter OS in elderly patients with cancer (Table 2).

**TABLE 2:** The univariate and multivariate analysis of SIPS in total patients and different tumor types.

Variables	OS (model 0)		OS (model 1)		OS (model 2)	
			
	Crude HR (95%CI)	Crude HR (95%CI)	Adjusted HR (95%CI)	Adjusted *P*	Adjusted HR (95%CI)	Adjusted *P*
**CRP**						
≤9.81	1		1		1	
>9.81	2.25 (1.96–2.57)	<0.001	1.84 (1.60–2.11)	<0.001	1.67 (1.44–1.92)	<0.001
**GNRI**						
≤93.85	1		1		1	
>93.85	2.05 (1.79–2.35)	<0.001	1.73 (1.49–2.01)	<0.001	1.67 (1.44–1.94)	<0.001
**ALI**						
≤23.49	1		1		1	
>23.49	2.23 (1.95–2.56)	<0.001	1.79 (1.55–2.06)	<0.001	1.57 (1.35–1.82)	<0.001
**LCR**						
≤2523.81	1		1		1	
>2523.81	2.37 (2.06–2.73)	<0.001	1.89 (1.63–2.18)	<0.001	1.69 (1.46–1.96)	<0.001
**SIPS**						
**Total patients**						
Low risk group (0)	1		1		1	
Medium risk group (1–2)	2.29 (1.90–2.77)	<0.001	1.92 (1.59–2.33)	<0.001	1.79 (1.47–2.17)	<0.001
High risk group (3–4)	3.71 (3.10–4.45)	<0.001	2.79 (2.30–3.38)	<0.001	2.40 (1.98–2.92)	<0.001
*P* for trend		<0.001		<0.001		<0.001
**Bytumor types *^a^***						
**Lung cancer**						
Low risk group (0)	1		1		1	
Medium risk group (1–2)	1.86 (1.40–2.48)	<0.001	1.47 (1.10–1.97)	0.009	1.42 (1.06–1.90)	0.019
High risk group (3–4)	2.68 (2.04–3.51)	<0.001	1.88 (1.40–2.51)	<0.001	1.72 (1.28–2.30)	<0.001
*P* for trend		<0.001		<0.001		<0.001
**Esophageal cancer**						
Low risk group (0)	1		1		1	
Medium risk group (1–2)	2.66 (1.28–5.55)	0.009	2.50 (1.13–5.53)	0.023	3.26 (1.40–7.59)	0.006
High risk group (3–4)	2.85 (1.34–6.03)	0.006	2.56 (1.16–5.67)	0.020	2.74 (1.16–6.47)	0.022
*P* for trend		0.010		0.042		0.058
**Gastric cancer**						
Low risk group (0)	1		1		1	
Medium risk group (1–2)	1.85 (1.15–2.96)	0.011	1.57 (0.97–2.55)	0.067	1.71 (1.04–2.81)	0.035
High risk group (3–4)	2.59 (1.64–4.08)	<0.001	1.91 (1.17–3.12)	0.010	2.34 (1.38–3.96)	0.002
*P* for trend		<0.001		0.011		0.002
**Colorectal cancer**						
Low risk group (0)	1		1		1	
Medium risk group (1–2)	2.66 (1.66–4.26)	<0.001	1.72 (1.05–2.80)	0.03	1.42 (0.85–2.39)	0.183
High risk group (3–4)	5.31 (3.38–8.36)	<0.001	3.93 (2.38–6.50)	<0.001	3.36 (1.96–5.75)	<0.001
*P* for trend		<0.001		<0.001		<0.001
**Other digestive cancers**						
Low risk group (0)	1		1		1	
Medium risk group (1–2)	2.09 (1.04–4.2)	0.039	1.78 (0.87–3.67)	0.116	1.45 (0.69–3.06)	0.332
High risk group (3–4)	4.43 (2.32–8.44)	<0.001	4.12 (2.11–8.05)	<0.001	3.57 (1.71–7.45)	0.001
*P* for trend		<0.001		<0.001		<0.001
**Other cancers**						
Low risk group (0)	1		1		1	
Medium risk group (1–2)	3.21 (1.66–6.21)	0.001	2.47 (1.26–4.87)	0.009	2.50 (1.22–5.13)	0.013
High risk group (3–4)	8.58 (4.70–15.66)	<0.001	6.37 (3.33–12.18)	<0.001	6.47 (3.23–12.95)	<0.001
*P* for trend		<0.001		<0.001		<0.001

*CRP: C-reactive protein; GNRI: geriatric nutrition risk index; ALI: advanced lung cancer inflammation index; LCR: lymphocyte to C-reactive protein ratio; SIPS: systemic inflammation prognostic score; HR, hazards ratio; CI, confidence interval; BMI: body mass index; KPS, karnofsky performance status; EORTC QLQ-C30: European Organization for Research and Treatment of Cancer Quality of Life Questionnaire-Core 30.Model 0: non-adjustment model.Model 1: adjusted for age, sex, tumor stage, and BMI.Model 2: adjusted for age, sex, tumor stage, BMI, tumor types, smoking, drinking, KPS, surgery, radiotherapy, chemotherapy, immunotherapy, nutritional intervention, EORTC QLQ-C30.By tumor types ^a^: adjusted for age, sex, tumor stage, BMI, smoking, drinking, KPS, surgery, radiotherapy, chemotherapy, immunotherapy, nutritional intervention, EORTC QLQ-C30.*

**FIGURE 3:**
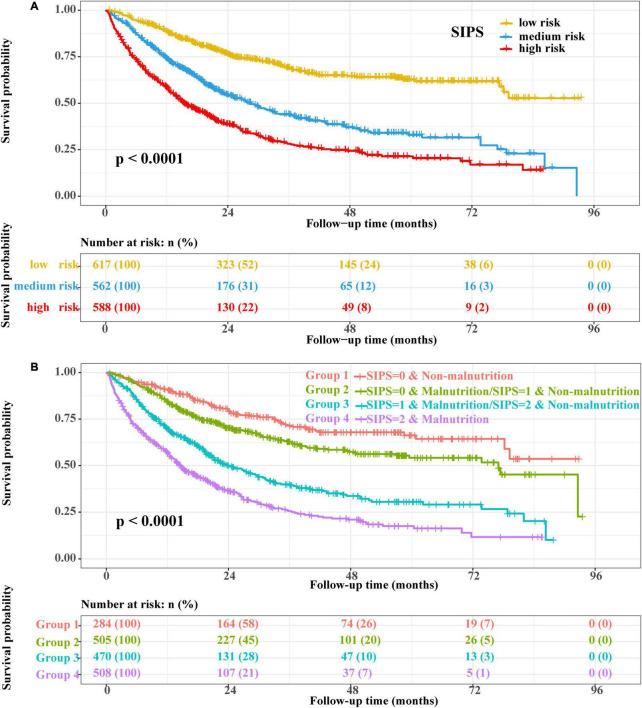
The Kaplan–Meier survival curves. **(A)** SIPS and **(B)** SIPS combined with PG-SGA. SIPS: systemic inflammation prognostic score; PG-SGA: patient-generated subjective global assessment.

**FIGURE 4:**
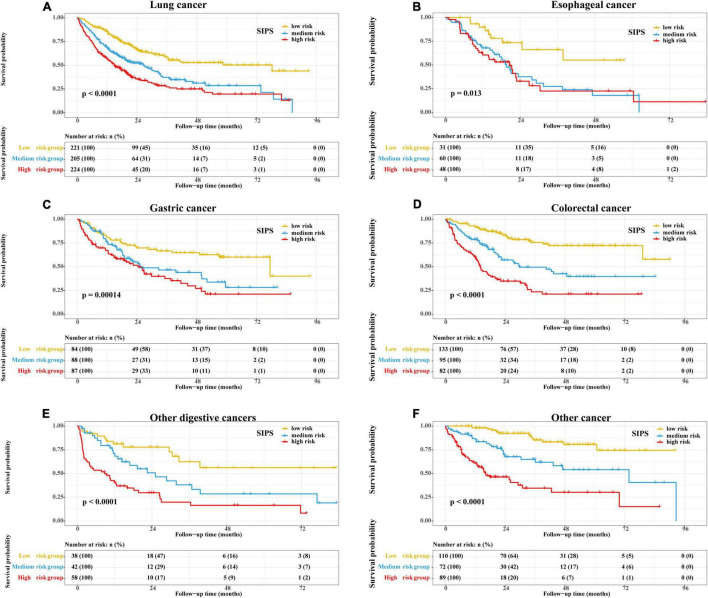
The Kaplan–Meier survival curves of SIPS stratified by different tumor types. **(A)** Lung cancer; **(B)** Esophageal cancer; **(C)** Gastric cancer; **(D)** Colorectal cancer; **(E)** Other digestive cancers; and **(F)** Other cancer. CRP: C-reactive protein; SIPS: systemic inflammation prognostic score.

Therefore, the inflammatory prognostic indices such as CRP, GNRI, ALI, and LCR were used to develop SIPS. The baseline population characteristics based on SIPS showed that age, sex, BMI, tumor stage, surgery, radiotherapy, chemotherapy, immunotherapy, KPS, EORTC QLQ-C30, smoking, PG-SGA, and nutritional interventions were significantly different between the groups (all *P* < 0.05) (Table 2). The number of patients in the high-risk and moderate-risk groups at different ages was higher than that in the low-risk groups (Figure 2C). We performed prognostic analysis on the constructed SIPS inflammation model and the univariate and multivariate survival analyzes showed that patients in the moderate-risk group (model 2: *P* < 0.001, HR = 1.79, 95% CI: 1.47–2.17) and the high-risk group (model 2: *P* < 0.001, HR = 2.40, 95% CI: 1.98–2.92) had shorter OS than those in the low-risk group (Table 3 and Figure 3A). In the survival analysis of different tumor subgroups, SIPS showed prognostic predictive values in lung cancer [*vs.* low-risk group, moderate-risk group (model 2: *P* = 0.019, HR = 1.42, 95% CI: 1.06–1.90), high-risk group (model 2: *P* < 0.001, HR = 1.72, 95% CI: 1.28–2.30)], esophageal cancer [*vs.* low-risk group, moderate-risk group (model 2: *P* = 0.006, HR = 3.26, 95% CI: 1.40–7.59), high-risk group (model 2: *P* = 0.022, HR = 2.74, 95% CI: 1.16–6.74)], gastric cancer [*vs.* low-risk group, moderate-risk group (model 2: *P* = 0.035, HR = 1.71, 95% CI: 1.04–2.81), high-risk group (model 2: *P* = 0.002, HR = 2.34, 95% CI: 1.38–3.96)], colorectal cancer [*vs.* low-risk group, moderate-risk group (model 2: *P* = 0.183, HR = 1.42, 95% CI: 0.85–2.39), high-risk group (model 2: *P* < 0.001, HR = 3.36, 95% CI: 1.96–5.75)], hepatobiliary pancreatic tumors [vs. low-risk group, moderate-risk group (model 2: *P* = 0.332, HR = 1.45, 95% CI: 0.69–3.06), high-risk group (model 2: *P* = 0.001, HR = 3.57, 95% CI: 1.71–7.45)], breast cancer [*vs.* low-risk group, moderate-risk group (model 2: *P* = 0.036, HR = 6.12, 95% CI: 1.13–33.13), high-risk group (model 2: *P* = 0.002, HR = 13.65, 95% CI: 2.63–70.84)], and other tumors [vs. low-risk group, moderate-risk group (model 2: *P* = 0.142, HR = 2.06, 95% CI: 0.78–5.38), high-risk group (model 2: *P* < 0.001, HR = 7.10, 95% CI: 2.89–17.47)] (Figure 4 and Table 3). The sensitivity analysis showed a similar result (Supplementary Table 2).

**TABLE 3:** Baseline characteristics stratified by SIPS.

Variables	SIPS group (n = 1767)			*P*-value
	
	Low risk (n = 617)	Medium risk (n = 562)	High risk (n = 588)	
Gender (%)				0.001
Male	379 (61.4)	369 (65.7)	422 (71.8)	
Female	238 (38.6)	193 (34.3)	166 (28.2)	
Age [mean (SD)]	70.09 (5.26)	71.25 (5.57)	71.63 (5.55)	<0.001
Age, > 70 years (%)	213 (34.5)	259 (46.1)	289 (49.1)	<0.001
BMI [mean (SD)]	23.64 (3.18)	22.17 (3.59)	20.90 (3.25)	<0.001
BMI, kg/m^2^ (%)				<0.001
<18.5	24 (3.9)	94 (16.7)	142 (24.1)	
18.5-23.9	325 (52.7)	294 (52.3)	344 (58.5)	
24-27.9	218 (35.3)	141 (25.1)	88 (15.0)	
≥ 28	50 (8.1)	33 (5.9)	14 (2.4)	
Smoking, yes (%)	291 (47.2)	290 (51.6)	336 (57.1)	0.002
Alcohol, yes (%)	131 (21.2)	143 (25.4)	153 (26.0)	0.105
Tumor types (%)				<0.001
Lung cancer	221 (35.8)	205 (36.5)	224 (38.1)	
Gastric cancer	84 (13.6)	88 (15.7)	87 (14.8)	
Esophageal cancer	31 (5.0)	60 (10.7)	48 (8.2)	
Colorectal cancer	133 (21.6)	95 (16.9)	82 (13.9)	
Other digestive cancers	38 (6.2)	42 (7.5)	58 (9.9)	
Breast cancer	46 (7.5)	16 (2.8)	9 (1.5)	
Female reproductive cancer	7 (1.1)	10 (1.8)	13 (2.2)	
Urological cancer	30 (4.9)	26 (4.6)	31 (5.3)	
Nasopharyngeal cancer	9 (1.5)	8 (1.4)	7 (1.2)	
Other cancer	18 (2.9)	12 (2.1)	29 (4.9)	
Tumor stage (%)				<0.001
I	62 (10.0)	41 (7.3)	26 (4.4)	
II	135 (21.9)	86 (15.3)	69 (11.7)	
III	208 (33.7)	143 (25.4)	128 (21.8)	
IV	212 (34.4)	292 (52.0)	365 (62.1)	
Surgery, yes (%)	324 (52.5)	235 (41.8)	203 (34.5)	<0.001
Radiotherapy, yes (%)	38 (6.2)	55 (9.8)	61 (10.4)	0.019
Chemotherapy, yes (%)	350 (56.7)	311 (55.3)	289 (49.1)	0.021
Immunotherapy, yes, (%)	40 (6.5)	22 (3.9)	12 (2.0)	0.001
PGSGA [mean (SD)]				<0.001
PGSGA (%)	284 (46.0)	172 (30.6)	80 (13.6)	
Well nourished	333 (54.0)	390 (69.4)	508 (86.4)	
Malnourished	5.17 (3.76)	7.14 (4.90)	9.78 (5.21)	<0.001
Nutritional intervention, yes (%)	106 (17.2)	123 (21.9)	171 (29.1)	<0.001
EORTCQLQ-C30 [mean (SD)]	46.84 (9.95)	50.48 (11.54)	54.36 (12.82)	<0.001
KPS [mean (SD)]	87.23 (9.73)	82.81 (13.68)	76.85 (16.58)	<0.001
KPS, < 60 (%)	13 (2.1)	51 (9.1)	105 (17.9)	<0.001

*SIPS: systemic inflammation prognostic score; SD: standard deviation; IQR: interquartile range; BMI: body mass index; ECOG PS: eastern cooperative oncology group performance status; KPS: karnofsky performance status; PGSGA: patient-generated subjective global assessment; EORTC QLQ-C30: The European Organization for Research and Treatment of Cancer (EORTC), Quality of Life Questionnaire-Core 30 (QLQ-C30).*

### Comparison and Validation of the Survival Prediction Ability of Systemic Inflammation Prognostic Score

**FIGURE 5:**
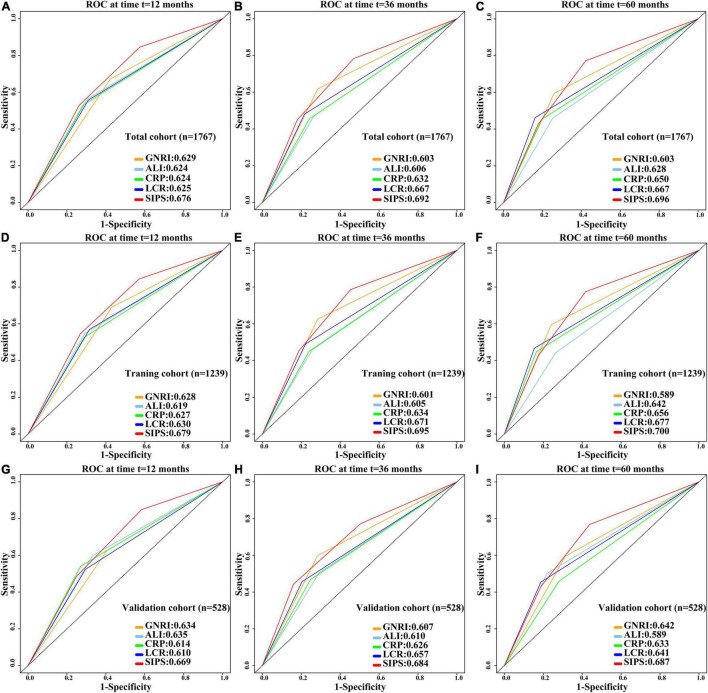
Comparison of 1-, 3-, and 5-year prognostic ROC for SIPS, CRP, GNRI, ALI, and LCR in the total cohort, training cohort, and validation cohort. **(A–C)** The 1-, 3-, and 5-year prognostic ROC in the total cohort; **(D–F)** The 1-, 3-, and 5-year prognostic ROC in the training cohort; **(G–I)**. The 1-, 3-, and 5-year prognostic ROC in the validation cohort. ROC: receiver operating characteristic curve; SIPS: systemic inflammation prognostic score; CRP: C-reactive protein; GNRI: geriatric nutrition risk index; ALI: advanced lung cancer inflammation index; LCR: lymphocyte to C-reactive protein ratio.

Systemic inflammation prognostic score (SIPS), CRP, GNRI, ALI, and LCR were compared. Prognostic ROC results at 1 year (SIPS: 0.676, CRP: 0.624, GNRI: 0.629, ALI: 0.624, and LCR: 0.625), 3 years (SIPS: 0.692, CRP: 0.632, GNRI: 0.603, ALI: 0.606, and LCR: 0.667), and 5 years (SIPS: 0.696, CRP: 0.650, GNRI:0.603; ALI: 0.628, and LCR: 0.667) showed that the area under the curve (AUC) of SIPS was superior to other biomarkers (Figures 5A–C). Similarly, the C-index of SIPS (0.649) was higher than that of CRP (0.594, comparative *P* < 0.001), GNRI (0.603, comparative *P* < 0.001), ALI (0.604, comparative *P* < 0.001), and LCR (0.609, comparative *P* < 0.001).

To further investigate the prognostic value of SIPS in elderly patients with cancer, the total cohort was randomly divided into the training (*n* = 1,239) and validation (*n* = 528) cohorts. The demographic and clinical features were similar between both the cohorts (Supplementary Table 3). In the training cohort, patients in the moderate-risk group (model 2: *P* < 0.001, HR = 1.93, 95% CI: 1.54–2.44) and high-risk group (model 2: *P* < 0.001, HR = 2.34, 95% CI: 1.86–2.96) had worse OS than those in the low-risk group (Supplementary Figure 4A and Supplementary Table 4). Prognostic ROC results at 1 year (SIPS: 0.679, CRP: 0.627, GNRI: 0.628, ALI: 0.619, and LCR: 0.630), 3 years (SIPS: 0.695, CRP: 0.634, GNRI: 0.601, ALI: 0.605, and LCR: 0.671), and 5 years (SIPS: 0.700, CRP: 0.656, GNRI: 0.589, ALI: 0.642, and LCR: 0.677) showed that the AUC of SIPS was superior to that of CRP, GNRI, ALI, and LCR (Figures 5D–F). The C-index of SIPS (0.649) was higher than that of CRP (0.601, comparative *P* < 0.001), GNRI (0.591, comparative *P* < 0.001), ALI (0.598, comparative *P* < 0.001), and LCR (0.610, comparative *P* < 0.001). In the validation cohort, patients in the moderate-risk group (model 2: *P* = 0.028, HR = 1.50, 95% CI: 1.05–2.16) and high-risk group (model 2: *P* < 0.001, HR = 2.69, 95% CI: 1.85–3.91) had worse OS than those in the low-risk group (Supplementary Figure 4B and Supplementary Table 4). SIPS had the higher AUC at 1 year (SIPS, 0.669; CRP, 0.614; GNRI, 0.634; ALI, 0.635; and LCR, 0.610), 3 years (SIPS: 0.684, CRP: 0.626, GNRI: 0.607, ALI: 0.610, and LCR: 0.657), and 5 years (SIPS: 0.687, CRP: 0.633, GNRI: 0.642, ALI: 0.589, and LCR: 0.641) than CRP, GNRI, ALI, and LCR (Figures 5G–I). The C-index of SIPS (0.647) was higher than that of CRP (0.606, comparative *P* < 0.001), GNRI (0.603, comparative *P* < 0.001), ALI (0.617, comparative *P* < 0.001), and LCR (0.607, comparative *P* < 0.001).

### Combined Effect of Systemic Inflammation Prognostic Score and Malnutrition

The prognostic value of the PG-SGA suggested that patients with malnutrition (model 2: *P* < 0.001, HR = 1.47, 95% CI: 1.23–1.76) had a shorter OS than patients without malnutrition (Supplementary Figure 5 and Supplementary Table 5). The combined survival analyzes of SIPS and PG-SGA indicated that the mortality risk of patients in the high-risk group and patients with malnutrition was 2.81 times higher than patients in the low-risk group and those without malnutrition (*P* < 0.001, 95% CI: 2.13–3.7) (Figure 3B and Supplementary Table 5).

## Discussion

This prospective multicenter cohort study aimed to investigate and develop a novel inflammation scoring system that can more accurately predict survival in elderly patients with cancer, based on clinically relevant prognostic parameters of inflammation. Elderly patients with cancer are particularly prone to developing cachexia and are, therefore, at high risk of increased mortality (21). Elderly patients often experience satiety earlier and feel less hungry, thereby increasing the risk of malnutrition (22). Moreover, an age-related progressive loss of muscle mass, known as sarcopenia, is associated with altered metabolism and further reduces physical activity in older adults (23). The inflammatory state in patients with cancer can accelerate the development of cachexia, which can lead to muscle wasting and worsen the patient’s prognosis (24). Therefore, it is very important to clarify the specific biological characteristics of tumor progression for further risk stratification and individualized treatment.

Considering collinearity and correlation between different variables and indicators, we performed dimensionality reduction through the LASSO regression and the Pearson correlation analysis to alleviate the interference between variables. Our multivariate survival analysis with different adjustment models showed that CRP, GNRI, ALI, and LCR all had significant survival predictive values in elderly patients with cancer. Similarly, previous studies have shown that CRP (11), GNRI (12), ALI (13), and LCR (14) can predict the prognosis of patients with cancer. Therefore, we developed and constructed SIPS, which consisted of CRP, GNRI, ALI, and LCR. The results of SIPS in different age distributions showed that the number of elderly patients with cancer increased with age and the number of patients in the moderate-risk and high-risk groups also increased. Thus, age and inflammation in elderly patients with cancer showed a positive trend. Previous studies have shown that age of > 70 years is associated with increased peripheral blood IL-6 levels (25). Furthermore, we found that SIPS was an independent survival predictor for elderly patients with cancer. After multivariate adjustment, the survival analysis of different tumor types showed that SIPS still had a significant survival prediction ability in elderly patients with cancer. In the past few years, many studies have begun to shift their focus to the inflammatory status of patients with cancer, while many other studies have also begun to identify the best predictors of inflammation and survival in patients with cancer; however, the results are variable and controversial and, hence, were of limited clinical value (26–28). Indeed, a single indicator has limitations and does not fully reflect a patient’s immune-inflammatory status. Wang et al. constructed an inflammatory-nutritional prognostic score (INPS) to predict survival in patients with stage III gastric cancer receiving adjuvant chemotherapy. IPNS, which includes BMI, NLR, PLR, LMR, and prealbumin, was a good independent predictor of stage III gastric cancer (29). Similarly, Galizia et al. established a new prognostic tool for colorectal cancer, namely, the Naples Prognostic Score, which includes albumin, cholesterol, NLR, and LMR and found that it performed better than existing single indicators in predicting patient outcomes (30). In this study, we compared the prognostic discrimination ability of SIPS and its components (CRP, GNRI, ALI, and LCR) in elderly patients with cancer, based on common clinical indicators, and found that the survival prediction value of SIPS in elderly patients with cancer was significantly higher than that of any of its components. Furthermore, we randomly divided 1,747 patients into the training cohort and the validation cohort in a 7:3 ratio and found that SIPS had independent prognostic value in elderly patients with cancer. A poor cancer prognosis is determined by changes in acute-phase proteins (elevated CRP and hypoalbuminemia) and white blood cell counts (elevated neutrophil count and low lymphocyte count) of the systemic inflammatory response (31). Some studies have found that the immune score of tumor tissue and serum IL-6, IL-11, or CD4 + /CD8 + T cells can also reflect the immune-inflammatory state, but the method is costly, inconvenient, and difficult to apply in clinical practice (32). Therefore, combined inflammatory markers, including the common components mentioned above, were evaluated and found to be more predictive of inflammation and survival in elderly patients with cancer than single markers.

It is important to highlight the nutritional status of elderly patients with cancer. Our combined analysis of SIPS and PG-SGA found that patients with malnutrition with a high SIPS had 2.81 times higher risk of death than patients with a high SIPS alone. Tumor-related inflammation and malnutrition are common in patients with cancer and are closely related to tumor recurrence and progression (7). Cancer-related malnutrition can be caused by cancer-activated systemic inflammation (33). The extravasation of tumor-produced proinflammatory cytokines can trigger further systemic inflammatory responses (31). In turn, these proinflammatory cytokines disrupt the metabolism of carbohydrates, fats, and proteins throughout the body (34). Cytokines can also affect the neuroendocrine control of appetite, leading to anorexia, which, in turn, leads to weight loss, changes in body composition, and decreased bodily function (33). There is a consistent link between symptoms, the presence of inflammatory markers, and an upregulated immune response (35). Nutritional and metabolic disturbances are common in patients with advanced cancer and can lead to weight loss, reduced quality of life, and poor treatment outcomes (36). Malnutrition also impairs immune responses and compromises the host defense against cancer (37).

The major strength of this study is that this was a prospective multicenter study using the LASSO regression to filter the dimensions of indicators and to compare and validate the models we constructed. More importantly, the parameters we used to construct SIPS are routinely tested, cost-effective, and readily available in clinical practice, which makes SIPS a very valuable indicator for prognostic stratification and treatment optimization strategies. This study has some limitations. First, there might be heterogeneity among different tumors, the ability of our SIPS to discriminate between the moderate-risk and high-risk groups in subgroups with esophageal cancer was not high, which might be due to the different pathological subtypes and tumor locations of esophageal cancer. This result requires further verification. Second, the prognosis of the elderly population with cancer is complex and is easily affected by physical and environmental factors. Additional confounding factors that may affect the prognosis of patients need to be considered. Finally, although we performed an internal validation of the prognostic value of SIPS, our findings were not validated by independent data, precluding confirmation of external validity.

## Conclusion

This study revealed that SIPS can predict mortality and prognosis in elderly patients with cancer and may have important clinical implications as an efficient and cost-effective scoring system. Patients with malnutrition in the high-risk group (SIPS of 4 or 5) had a 2.81 times higher risk of death than patients without malnutrition in the low-risk group (SIPS of 0).

## Data Availability Statement

The raw data supporting the conclusions of this article will be made available by the authors, without undue reservation.

## Ethics Statement

The studies involving human participants were reviewed and approved by this study adhered to the tenets of the Declaration of Helsinki. All participants provided written informed consent, and this study was approved by the institutional review board of each hospital (registration number: ChiCTR1800020329). The patients/participants provided their written informed consent to participate in this study. Written informed consent was obtained from the individual(s) for the publication of any potentially identifiable images or data included in this article.

## Author Contributions

G-TR, wrote the manuscript. G-TR, H-LX, and LD analyzed and interpreted the patient data. G-TR, H-LX, LD, and H-PS made substantial contributions to the conception, design, and intellectual content of the study. All authors read and approved the final manuscript.

## Conflict of Interest

The authors declare that the research was conducted in the absence of any commercial or financial relationships that could be construed as a potential conflict of interest. The reviewer JG declared a shared affiliation with one of the author Y-ZGo to the handling editor at the time of review.

## Publisher’s Note

All claims expressed in this article are solely those of the authors and do not necessarily represent those of their affiliated organizations, or those of the publisher, the editors and the reviewers. Any product that may be evaluated in this article, or claim that may be made by its manufacturer, is not guaranteed or endorsed by the publisher.

## Footnotes

1
http://www.R-project.org

## Supplementary Material

Supplementary Figure 1 Flowchart of patient selection for this study.

Supplementary Figure 2 Optimal cutoff value of CRP, GNRI, ALI, and LCR according to the results of the standardized log-rank statistic. **(A)** CRP; **(B)** GNRI; **(C)** ALI; and **(D)** LCR. Notes: CRP: C-reactive protein; GNRI: geriatric nutrition risk index; ALI: advanced lung cancer inflammation index; LCR: lymphocyte to C-reactive protein ratio.

Supplementary Figure 3 The Kaplan–Meier survival curves of CRP, GNRI, ALI, and LCR. **(A)** CRP; **(B)** GNRI; **(C)** ALI; and **(D)** LCR. Notes: CRP: C-reactive protein; GNRI: geriatric nutrition risk index; ALI: advanced lung cancer inflammation index; LCR: lymphocyte to C-reactive protein ratio.

Supplementary Figure 4 The Kaplan–Meier survival curves of SIPS in the training cohort and the validation cohort. **(A)** The training cohort and **(B)** The validation cohort. Notes: SIPS: systemic inflammation prognostic score.

Supplementary Figure 5 The Kaplan–Meier survival curve of PGSGA. Notes: PGSGA: patient-generated subjective global assessment.
