# Supplementary material for: A Novel Systemic Inflammation Prognostic Score to Stratify Survival in Elderly Patients With Cancer
Source: Front Nutr. 2022 Jul 5;9:893753. doi: 10.3389/fnut.2022.893753 (PMC9294408; doi:10.3389/fnut.2022.893753)
Supplement: Supplementary file 1 [file Table_1.DOCX]

**Table S1 Abbreviations and cut-off values for CRP, ALI, GNRI, LCR**

| Variables | Full name | Cut-off values |
| --- | --- | --- |
| CRP | C-reactive protein | 9.81 |
| ALI | Advanced lung cancer inflammation index | 23.49 |
| GNRI | Geriatric nutrition risk index | 93.85 |
| LCR | Lymphocyte to C-reactive protein ratio | 2523.81 |
