# Supplementary material for: A Novel Systemic Inflammation Prognostic Score to Stratify Survival in Elderly Patients With Cancer
Source: Front Nutr. 2022 Jul 5;9:893753. doi: 10.3389/fnut.2022.893753 (PMC9294408; doi:10.3389/fnut.2022.893753)
Supplement: Supplementary file 2 [file Table_2.DOCX]

**Table S2 Sensitivity analysis of SIPS in total patients and different tumor types*.**

| Variables | OS (model 0) | |  | OS (model 2) | |
| --- | --- | --- | --- | --- | --- |
|  | Crude HR (95%CI) | Crude HR (95%CI) |  | Adjusted HR (95%CI) | Adjusted P |
| SIPS |  |  |  |  |  |
| Total patients |  |  |  |  |  |
| Low risk group (0) | 1 |  |  | 1 |  |
| Medium risk group (1-2) | 2.28 (1.85-2.82) | <0.001 |  | 1.88 (1.51-2.34) | <0.001 |
| High risk group (3-4) | 3.10 (2.51-3.82) | <0.001 |  | 2.13 (1.69-2.68) | <0.001 |
| *P* for trend |  | <0.001 |  |  | <0.001 |
| Lung cancer |  |  |  |  |  |
| Low risk group (0) | 1 |  |  | 1 |  |
| Medium risk group (1-2) | 1.77 (1.28-2.45) | 0.001 |  | 1.42 (1.02-1.99) | 0.039 |
| High risk group (3-4) | 2.30 (1.68-3.16) | <0.001 |  | 1.66 (1.18-2.35) | 0.004 |
| *P* for trend |  | 0.001 |  |  | 0.004 |
| Esophageal cancer |  |  |  |  |  |
| Low risk group (0) | 1 |  |  | 1 |  |
| Medium risk group (1-2) | 2.16 (1.01-4.61) | 0.046 |  | 1.99 (0.81-4.92) | 0.136 |
| High risk group (3-4) | 2.17 (0.99-4.76) | 0.054 |  | 2.81 (1.03-7.65) | 0.043 |
| p for trend |  | 0.004 |  |  | 0.040 |
| Gastric cancer |  |  |  |  |  |
| Low risk group (0) | 1 |  |  | 1 |  |
| Medium risk group (1-2) | 2.14 (1.25-3.65) | 0.005 |  | 2.07 (1.17-3.66) | 0.013 |
| High risk group (3-4) | 2.21 (1.28-3.83) | 0.005 |  | 1.75 (0.93-3.29) | 0.081 |
| *P* for trend |  | 0.071 |  |  | 0.082 |
| Colorectal cancer |  |  |  |  |  |
| Low risk group (0) | 1 |  |  | 1 |  |
| Medium risk group (1-2) | 2.69 (1.57-4.6) | <0.001 |  | 1.46 (0.80-2.67) | 0.213 |
| High risk group (3-4) | 4.74 (2.78-8.08) | <0.001 |  | 3.17 (1.68-5.99) | <0.001 |
| *P* for trend |  | 0.001 |  |  | <0.001 |
| Other digestive cancers |  |  |  |  |  |
| Low risk group (0) | 1 |  |  | 1 |  |
| Medium risk group (1-2) | 2.41 (1.08-5.37) | 0.032 |  | 1.99 (0.81-4.92) | 0.136 |
| High risk group (3-4) | 2.90 (1.28-6.53) | 0.010 |  | 2.81 (1.03-7.65) | 0.043 |
| *P* for trend |  | 0.010 |  |  | 0.040 |
| Other cancers |  |  |  |  |  |
| Low risk group (0) | 1 |  |  | 1 |  |
| Medium risk group (1-2) | 2.64 (1.32-5.27) | 0.006 |  | 2.39 (1.06-5.40) | 0.036 |
| High risk group (3-4) | 6.31 (3.32-11.99) | <0.001 |  | 6.05 (2.76-13.29) | <0.001 |
| *P* for trend |  | 0.001 |  |  | <0.001 |

Notes: * The sensitivity analysis was to exclude patients who died within 6 months. SIPS: systemic inflammation prognostic score; HR, hazards ratio; CI, confidence interval; BMI: body mass index; KPS, karnofsky performance status; EORTC QLQ-C30: European Organization for Research and Treatment of Cancer Quality of Life Questionnaire-Core 30.

Model 0: non-adjustment model.

Model 2: adjusted for age, sex, tumor stage, BMI, tumor types, smoking, drinking, KPS, surgery, radiotherapy, chemotherapy, immunotherapy, nutritional intervention, EORTC QLQ-C30.
