# Supplementary material for: A Novel Systemic Inflammation Prognostic Score to Stratify Survival in Elderly Patients With Cancer
Source: Front Nutr. 2022 Jul 5;9:893753. doi: 10.3389/fnut.2022.893753 (PMC9294408; doi:10.3389/fnut.2022.893753)
Supplement: Supplementary file 3 [file Table_3.DOCX]

**Table S3 Baseline characteristics of the training cohort and validation cohort.**

| Variables | Training cohort | Validation cohort | *P* value |
| --- | --- | --- | --- |
|  | (n=1239) | (n=528) |  |
| Gender (%) |  |  | 0.733 |
| Male | 824 (66.5) | 346 (65.5) |  |
| Female | 415 (33.5) | 182 (34.5) |  |
| Age (mean (SD)) | 70.92 (5.51) | 71.11 (5.45) | 0.500 |
| Age, >70 years (%) | 527 (42.5) | 234 (44.3) | 0.522 |
| BMI (mean (SD)) | 22.31 (3.51) | 22.13 (3.56) | 0.306 |
| BMI, kg/m^2^ (%) |  |  | 0.610 |
| <18.5 | 178 (14.4) | 82 (15.5) |  |
| 18.5-23.9 | 671 (54.2) | 292 (55.3) |  |
| 24-27.9 | 324 (26.2) | 123 (23.3) |  |
| ≥28 | 66 (5.3) | 31 (5.9) |  |
| Smoking, yes (%) | 642 (51.8) | 275 (52.1) | 0.959 |
| Alcohol, yes (%) | 315 (25.4) | 112 (21.2) | 0.067 |
| Tumor types (%) |  |  | 0.327 |
| Lung cancer | 466 (37.6) | 184 (34.8) |  |
| Gastric cancer | 169 (13.6) | 90 (17.0) |  |
| Esophageal cancer | 97 (7.8) | 42 (8.0) |  |
| Colorectal cancer | 215 (17.4) | 95 (18.0) |  |
| Other digestive cancers | 100 (8.1) | 38 (7.2) |  |
| Breast cancer | 50 (4.0) | 21 (4.0) |  |
| Female reproductive cancer | 22 (1.8) | 8 (1.5) |  |
| Urological cancer | 55 (4.4) | 32 (6.1) |  |
| Nasopharyngeal cancer | 21 (1.7) | 3 (0.6) |  |
| Other cancer | 44 (3.6) | 15 (2.8) |  |
| Tumor stage (%) |  |  | 0.228 |
| I | 89 (7.2) | 40 (7.6) |  |
| II | 217 (17.5) | 73 (13.8) |  |
| III | 325 (26.2) | 154 (29.2) |  |
| IV | 608 (49.1) | 261 (49.4) |  |
| Surgery, yes (%) | 530 (42.8) | 232 (43.9) | 0.690 |
| Radiotherapy, yes (%) | 108 (8.7) | 46 (8.7) | 1.000 |
| Chemotherapy, yes (%) | 677 (54.6) | 273 (51.7) | 0.280 |
| Immunotherapy, yes, (%) | 54 (4.4) | 20 (3.8) | 0.676 |
| PGSGA (mean (SD)) | 7.27 (5.05) | 7.48 (4.96) | 0.419 |
| PGSGA (%) |  |  | 0.763 |
| Well nourished | 379 (30.6) | 157 (29.7) |  |
| Malnourished | 860 (69.4) | 371 (70.3) |  |
| Nutritional intervention, yes (%) | 265 (21.4) | 135 (25.6) | 0.063 |
| EORTCQLQ-C30 (mean (SD)) | 50.68 (11.65) | 50.07 (12.40) | 0.324 |
| KPS (mean (SD)) | 82.29 (14.05) | 82.56 (14.62) | 0.720 |
| KPS, <60 (%) | 122 (9.8) | 47 (8.9) | 0.596 |

Notes: SD: standard deviation; IQR: interquartile range; BMI: body mass index; ECOG PS: eastern cooperative oncology group performance status; KPS: karnofsky performance status; PGSGA: patient-generated subjective global assessment; EORTC QLQ-C30: The European Organization for Research and Treatment of Cancer (EORTC), Quality of Life Questionnaire-Core 30 (QLQ-C30).
