# Supplementary material for: A Novel Systemic Inflammation Prognostic Score to Stratify Survival in Elderly Patients With Cancer
Source: Front Nutr. 2022 Jul 5;9:893753. doi: 10.3389/fnut.2022.893753 (PMC9294408; doi:10.3389/fnut.2022.893753)
Supplement: Supplementary file 4 [file Table_4.DOCX]

**Table S4 Survival analysis of SIPS in the training cohort and validation cohort.**

| Variables | OS (model 0) | |  | OS (model 2) | |
| --- | --- | --- | --- | --- | --- |
|  | Crude HR (95%CI) | Crude HR (95%CI) |  | Adjusted HR (95%CI) | Adjusted P |
| SIPS |  |  |  |  |  |
| Training cohort |  |  |  |  |  |
| Low risk group (0) | 1 |  |  | 1 |  |
| Medium risk group (1-2) | 2.49 (1.99-3.12) | <0.001 |  | 1.93 (1.54-2.44) | <0.001 |
| High risk group (3-4) | 3.70 (2.98-4.58) | <0.001 |  | 2.34 (1.86-2.96) | <0.001 |
| *P* for trend |  | <0.001 |  |  | <0.001 |
| Validation cohort |  |  |  |  |  |
| Low risk group (0) |  |  |  |  |  |
| Medium risk group (1-2) | 1.85 (1.31-2.62) | <0.001 |  | 1.50 (1.05-2.16) | 0.028 |
| High risk group (3-4) | 3.69 (2.65-5.14) | <0.001 |  | 2.69 (1.85-3.91) | <0.001 |
| *P* for trend |  | <0.001 |  |  | <0.001 |

Notes: SIPS: systemic inflammation prognostic score; HR, hazards ratio; CI, confidence interval; BMI: body mass index; KPS, karnofsky performance status; EORTC QLQ-C30: European Organization for Research and Treatment of Cancer Quality of Life Questionnaire-Core 30.

Model 0: non-adjustment model.

Model 2: adjusted for age, sex, tumor stage, BMI, tumor types, smoking, drinking, KPS, surgery, radiotherapy, chemotherapy, immunotherapy, nutritional intervention, EORTC QLQ-C30.
