# Supplementary material for: A Novel Systemic Inflammation Prognostic Score to Stratify Survival in Elderly Patients With Cancer
Source: Front Nutr. 2022 Jul 5;9:893753. doi: 10.3389/fnut.2022.893753 (PMC9294408; doi:10.3389/fnut.2022.893753)
Supplement: Supplementary file 5 [file Table_5.DOCX]

**Table S5 Combined analysis**

| Variables | OS (model 0) | |  | OS (model 2) | |
| --- | --- | --- | --- | --- | --- |
|  | Crude HR (95%CI) | Crude *P* |  | Adjusted HR (95%CI) | Adjusted *P* |
| PGSGA |  |  |  |  |  |
| Non- malnutrition | 1 |  |  | 1 |  |
| Malnutrition | 1.97 (1.67-2.32) | <0.001 |  | 1.47 (1.23-1.76) | <0.001 |
| SIPS&PGSGA |  |  |  |  |  |
| SIPS=0 & Non-malnutrition | 1 |  |  | 1 |  |
| SIPS=0 & Malnutrition/SIPS=1 & Non-malnutrition | 1.48 (1.13-1.95) | 0.005 |  | 1.29 (0.98-1.70) | 0.073 |
| SIPS=1 & Malnutrition/SIPS=2 & Non-malnutrition | 2.92 (2.25-3.79) | <0.001 |  | 2.11 (1.60-2.77) | <0.001 |
| SIPS=2 & Malnutrition | 4.61 (3.58-5.94) | <0.001 |  | 2.81 (2.13-3.70) | <0.001 |
| p for trend |  | <0.001 |  |  | <0.001 |

Notes: CRP: C-reactive protein; LHR: LDL-c/HDL-c ratio; HDL-c: high-density lipoprotein cholesterol; LDL-c: low-density lipoprotein cholesterol; HR, hazards ratio; CI, confidence interval; BMI: body mass index; KPS, karnofsky performance status.

Model 0: Unadjusted.

Model 2: adjusted for age, sex, tumor stage, BMI, tumor types, smoking, drinking, KPS, surgery, radiotherapy, chemotherapy, immunotherapy, nutritional intervention, EORTC QLQ-C30.
