# Supplementary figures and images for: A Novel Systemic Inflammation Prognostic Score to Stratify Survival in Elderly Patients With Cancer
Source: Front Nutr. 2022 Jul 5;9:893753. doi: 10.3389/fnut.2022.893753 (PMC9294408; doi:10.3389/fnut.2022.893753)

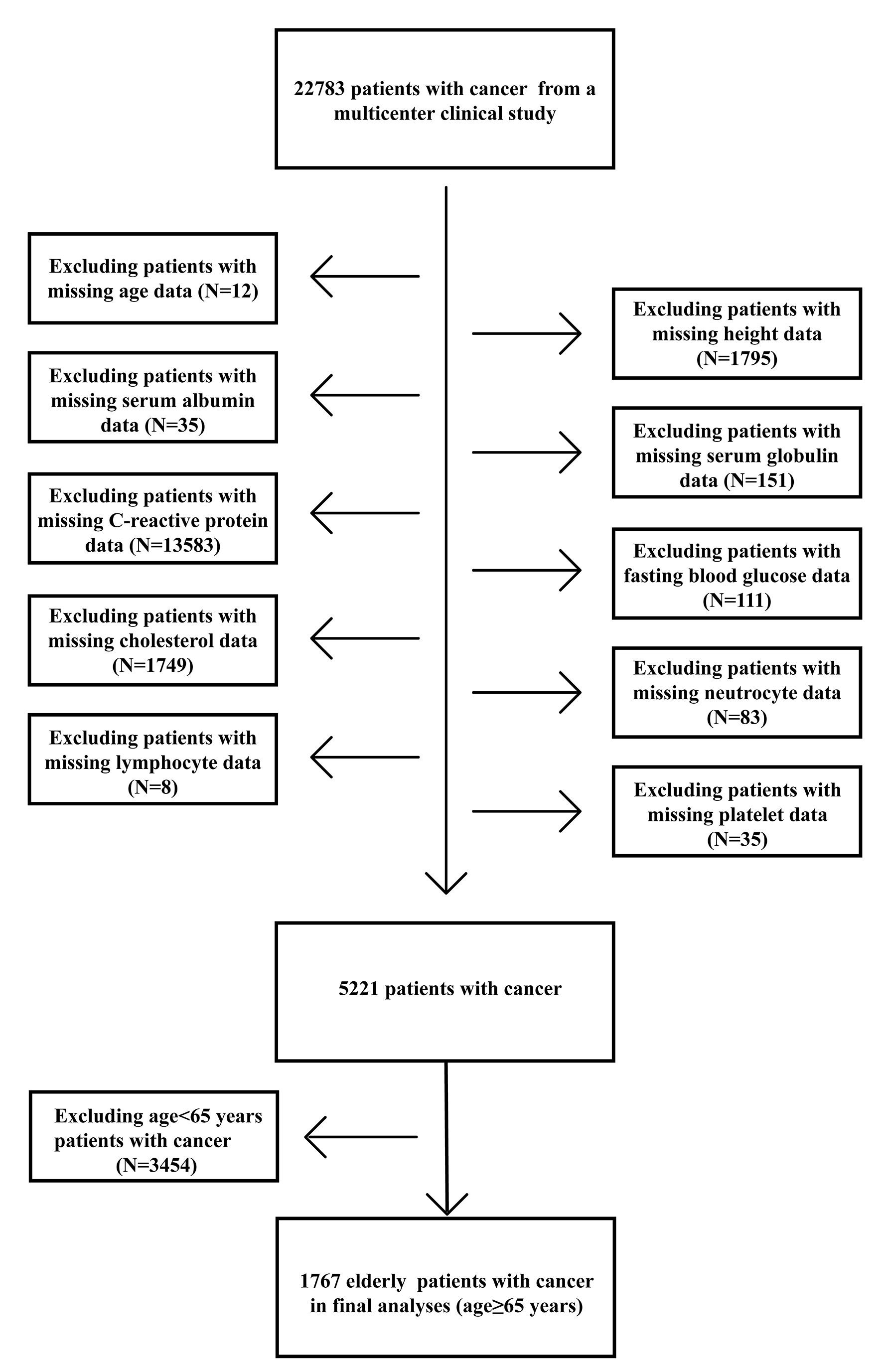

Supplement: Supplementary Figure 1 — Flowchart of patient selection for this study. [file Image_1.TIF]

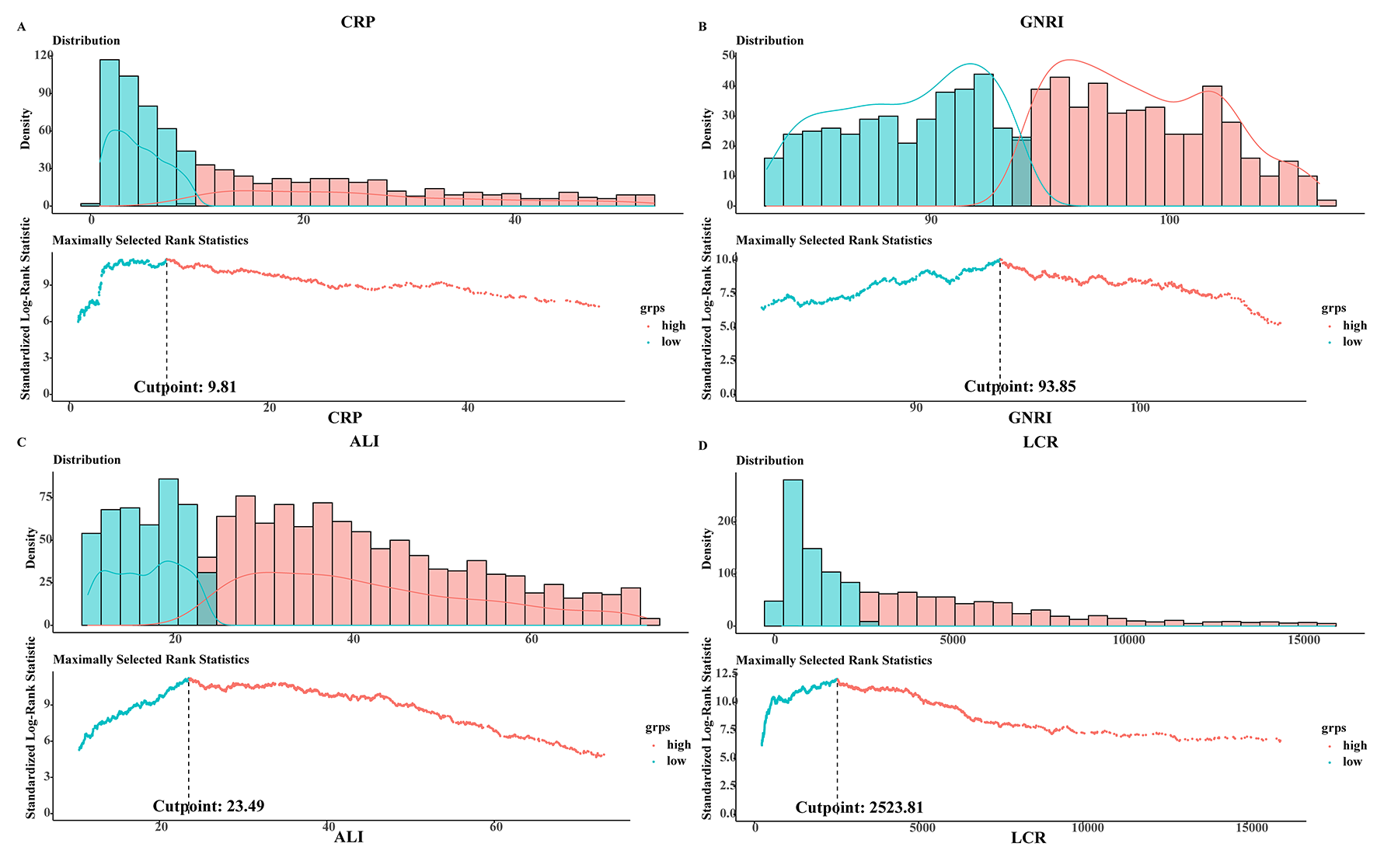

Supplement: Supplementary Figure 2 — Optimal cutoff value of CRP, GNRI, ALI, and LCR according to the results of the standardized log-rank statistic. (A) CRP; (B) GNRI; (C) ALI; and (D) LCR. Notes: CRP: C-reactive protein; GNRI: geriatric nutrition risk index; ALI: advanced lung cancer inflammation index; LCR: lymphocyte to C-reactive protein ratio. [file Image_2.TIF]

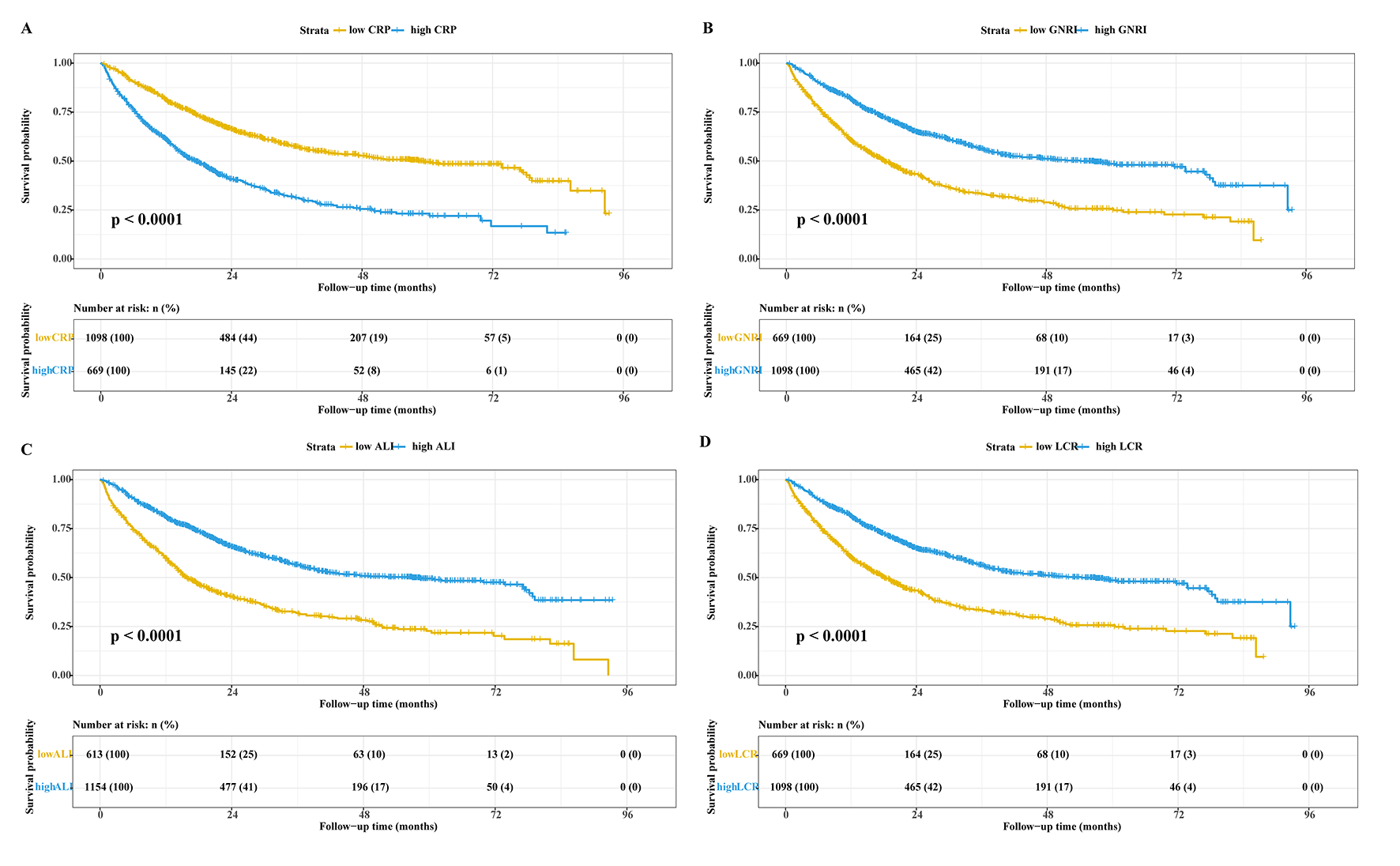

Supplement: Supplementary Figure 3 — The Kaplan–Meier survival curves of CRP, GNRI, ALI, and LCR. (A) CRP; (B) GNRI; (C) ALI; and (D) LCR. Notes: CRP: C-reactive protein; GNRI: geriatric nutrition risk index; ALI: advanced lung cancer inflammation index; LCR: lymphocyte to C-reactive protein ratio. [file Image_3.TIF]

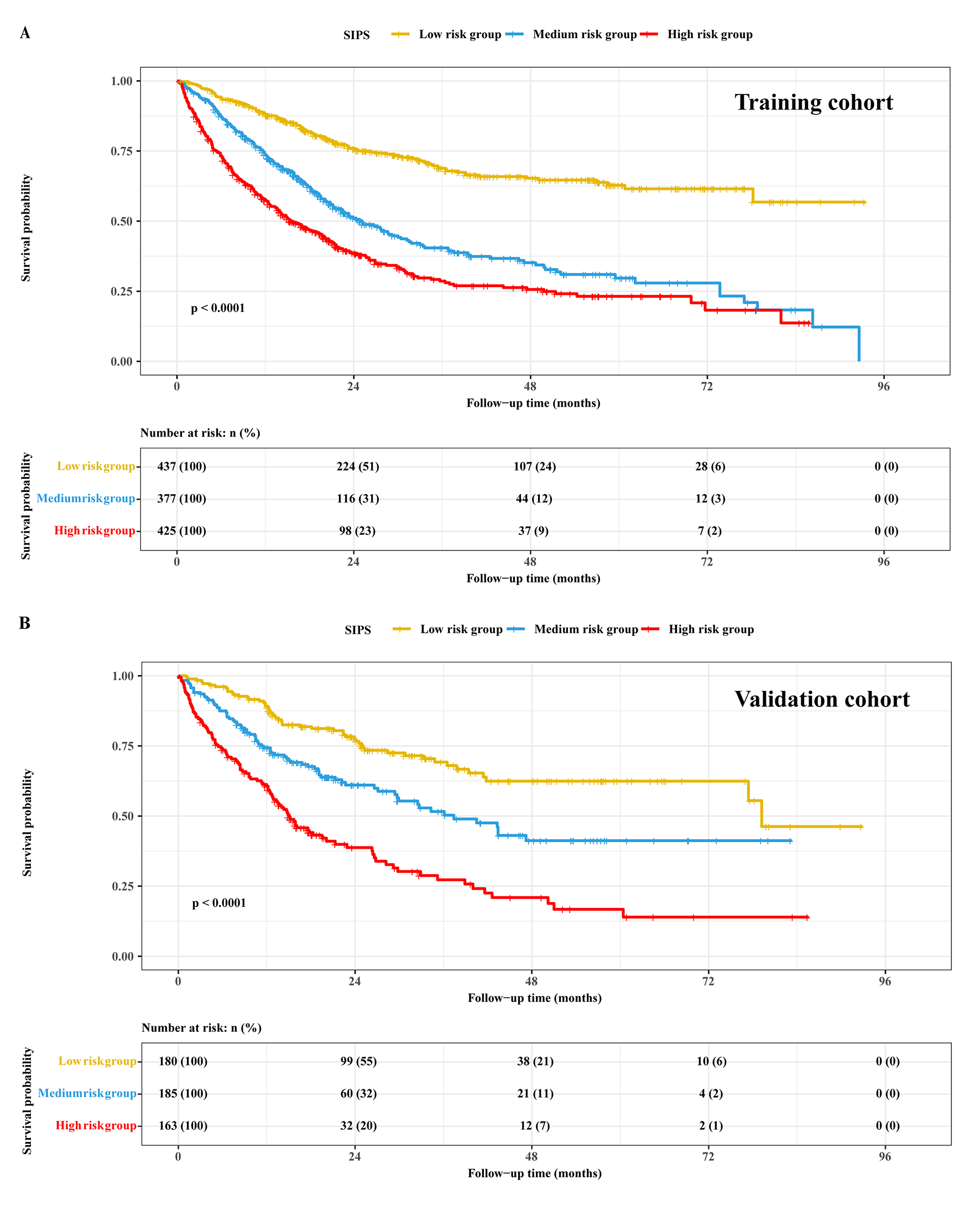

Supplement: Supplementary Figure 4 — The Kaplan–Meier survival curves of SIPS in the training cohort and the validation cohort. (A) The training cohort and (B) The validation cohort. Notes: SIPS: systemic inflammation prognostic score. [file Image_4.TIF]

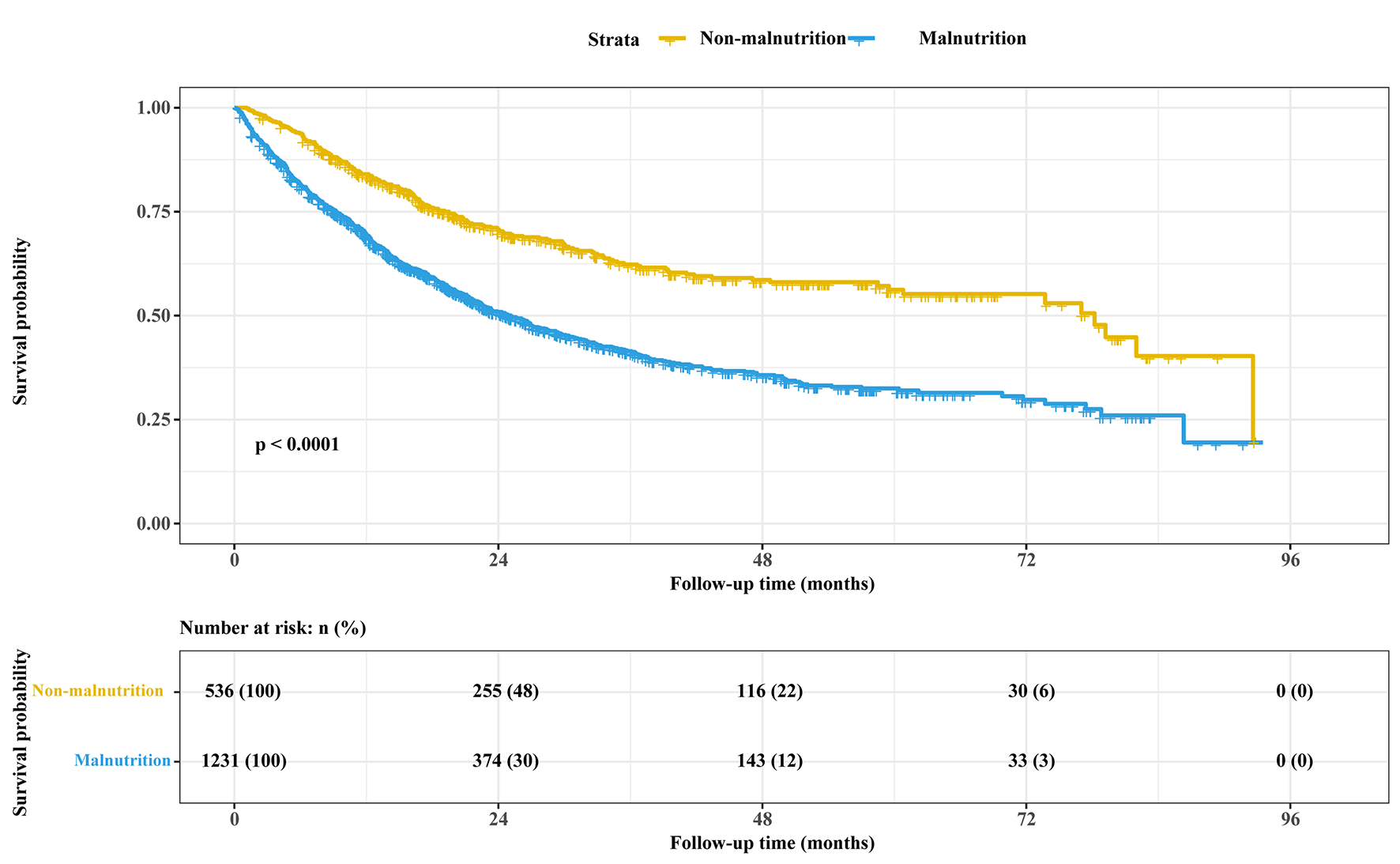

Supplement: Supplementary Figure 5 — The Kaplan–Meier survival curve of PGSGA. Notes: PGSGA: patient-generated subjective global assessment. [file Image_5.TIF]
